# 

**Published:** 1895-12

**Authors:** 


### BOOKS RECEIVED.

The Principles and Practice of Medicine. Designed for the use of Practitioners and Students of Medicine by William Osler, M. D., Fellow of the Royal College of Physicians, London ; Professor of Medicine in the Johns Hopkins University and Physician-in-chief to the Johns Hopkins Hospital, Baltimore, etc., etc. Octavo, pp. xvi.—1,143. Second edition. New York : D. Appleton & Co. 1895.

An American Text-book of Obstetrics for Practitioners and Students. Richard C. Norris, M. D., Editor, Robert L. Dickinson, Art Editor. Royal 8vo, pp. 1009. With nearly 900 colored and half-tone illustrations. Price, $7, cloth ; $8, sheep ; $9, one-half Russia. Philadelphia : W. B. Saunders, 925 Walnut street. 1895.

Dunglison's Dictionary of Medical Science. Twenty-first Edition, with Appendix. By Robley Dunglison, M. D., LL.D., late Professor of Institutes of Medicine in the Jefferson Medical College of Philadelphia. Edited by Richard J. Dunglison, A. M., M. D. New (21st) edition, thoroughly revised, greatly enlarged and improved, with the pronunciation, accentuation and derivation of the terms. In one magnificent imperial 8vo volume of 1225 pages. Cloth, $7 ; leather, $8. Thumb-letter index for quick use, 75 cents extra. Philadelphia : Lea Brothers & Co., Publishers. 1895.

Functional and Organic Diseases of the Stomach. By Sidney Martin, M. D., F. R. S., F. R. C. P., Assistant Physician and Assistant Professor of Clinical Medicine at University College Hospital ; Assistant Physician to the Hospital for Consumption and Diseases of the Chest, Brompton. Octavo, pp. xvi.—505. With fifty-seven illustrations. Edinburgh and London : Young J. Pentland. Philadelphia : J. B. Lippincott Co. 1895.

Transactions of the State Medical Society of Wisconsin for the Year 1895. Volume XXIX. Constitution and by-laws and list of members. Charles S. Sheldon, M. D., secretary. Madison, Wisconsin : Tracy, Gibbs & Co., Printers and Stereotypers. 1895.

Transactions of the Colorado State Medical Society. Twenty-fifth annual convention, held in Denver, Col., June 18, 19, 20, 1895. By-laws and list of members. Edwin R. Axtell, M. D., secretary. Denver, Col.: Published for the Society.

New York State Commission in Lunacy. Sixth Annual Report, October 1, 1893, to September 30, 1894. T. E. McGarr, secretary. Transmitted to the legislature May 24, 1895. Albany : James B. Lyon, State Printer. 1895.

A Manual of Operative Surgery. By Lewis A. Stimson, B. A., M. D., Professor of Clinical Surgery in the University of the City of New York. New (3d) edition. In one royal 12mo volume of 614 pages, with 306 illustrations. Cloth, $3.75. Philadelphia : Lea Brothers & Co., Publishers. 1895.

Pediatrics, the Hygienic and Medical Treatment of Children. By Thomas Morgan Rotch, M. D., Professor of the Diseases of Children, Harvard University. Royal 8vo, pp. xii.—1,124. Illustrated. Philadelphia : J. B. Lippincott Company. 1895.

Supplement to the International Encyclopedia of Surgery. Edited by John Ashhurst, Jr., M. D., LL. D., Philadelphia. One royal 8vo volume, of 1,136 pages, illustrated by numerous wood-engravings and a chromo-lithographic plate. Cloth, $7.50 ; leather, $8.50. To subscribers to the entire set, cloth, $6; leather, $7, and half morocco, $8. New York: William Wood & Co. 1895.

Pregnancy, Labor and the Puerperal State. By Egbert H. Grandin, M. D., Consulting Surgeon to the New York Maternity Hospital; Consulting Gynecologist to the French Hospital, New York, etc.; and George W. Jarman, M. D., Obstetric Surgeon to the New York Maternity Hospital; Gynecologist to the Cancer Hospital, New York, etc. Illustrated, with forty-one (41) original full-page photographic plates from nature. Royal 8vo, pp. viii.—261. Cloth, $2.50 net. Philadelphia: The F. A. Davis Co., Publishers, 1914 and 1916 Cherry street. 1895.

Manual of Gynecology. By Henry T. Byford, Professor of Gynecology and Clinical Gynecology in the College of Physicians and Surgeons of Chicago; Professor of Clinical Gynecology in the Woman's Medical School of Northwestern University; Professor of Gynecology in the Post-Graduate Medical School of Chicago. Octavo, pp. xii.—488, containing 234 illustrations, many of which are original. Price, $2.50. Philadelphia: P. Blakiston, Son & Co., 1012 Walnut street. 1895.

Handbook of the Diagnosis and Treatment of Skin Diseases. By Arthur Van Harlingen, Ph. B. (Yale), M. D., Emeritus Professor of Dermatology in the Philadelphia Polyclinic; Dermatologist to the Howard Hospital. Third edition, enlarged and revised. Small 8vo, pp. xvi.—577. Sixty illustrations, several of which are in colors. Price, $2.75. Philadelphia: P. Blakiston, Son & Co., 1012 Walnut street. 1895.

A Manual of Syphilis and the Venereal Diseases. By James Nevins Hyde, A. M., M. D., Professor of Skin and Venereal Diseases, Rush Medical College; Dermatologist to the Presbyterian, Michael Reese and Augustana Hospitals and Consulting Physician to the Hospital for Women and Children, Chicago; and Frank H. Montgomery, M. D., Lecturer on Dermatology and Genito-Urinary Diseases and Chief Assistant to the Clinic for Skin and Venereal Diseases, Rush Medical College; Attending Physician for Skin and Venereal Diseases, St. Elizabeth Hospital, Chicago. Small 8vo, pp. 618. With forty-four illustrations in the text and eight full-page plates in colors and tints. Price, $2.50. Philadelphia: W. B. Saunders, 925 Walnut street. 1895.

Materia Medica and Therapeutics. A Practical Treatise, with Especial Reference to the Clinical Application of Drugs. By John V. Shoemaker, A. M., M. D., LL. D., Professor of Materia Medica, Pharmacology, Therapeutics and Clinical Medicine, and Clinical Professor of Diseases of the Skin in the Medico-Chirurgical College of Philadelphia; Physician to the Medico-Chirurgical Hospital, Philadelphia, etc., etc. Third edition, thoroughly revised. Reset with new type and printed from new electrotype plates. Royal 8vo, pp. ix.—1,108. Extra cloth, $5 net; sheep, $5.75 net. Philadelphia: The F. A. Davis Co., Publishers, 1914 and 1916 Cherry Street. 1895.

Practical Urinalysis and Urinary Diagnosis. A Manual for the Use of Physicians, Surgeons and Students. By Charles W. Purdy, M. D., Queen's University; Fellow of the Royal College of Physicians and Surgeons, Kingston; Professor of Urology and Urinary Diagnosis at the Chicago Post-Graduate Medical School. Second revised edition. With numerous illustrations, including photo-engravings and colored plates. In one crown 8vo volume, 360 pages. In extra cloth, $2.50 net. Philadelphla: The F. A. Davis Co., Publishers, 1914 and 1916 Cherry street. 1895.

